# Factors influencing agency nursing and moonlighting among nurses in South Africa

**DOI:** 10.3402/gha.v7.23585

**Published:** 2014-03-18

**Authors:** Laetitia C. Rispel, Duane Blaauw, Tobias Chirwa, Katinka de Wet

**Affiliations:** 1 Centre for Health Policy & Medical Research Council Health Policy Research Group, School of Public Health, Faculty of Health Sciences, University of the Witwatersrand, Johannesburg, South Africa; 2 Division of Epidemiology and Biostatistics, School of Public Health, Faculty of Health Sciences, University of the Witwatersrand, Johannesburg, South Africa; 3 Department of Sociology, University of the Free State, Bloemfontein, South Africa

**Keywords:** moonlighting, agency nursing, nurses, human resources, overtime, South Africa

## Abstract

**Background:**

In South Africa, nurses are the largest category of the health care providers. Their optimal performance is critical for the successful implementation of impending health sector reforms.

**Objective:**

This paper examines the occurrence of agency nursing, moonlighting, and overtime among nurses in South Africa, and the factors influencing moonlighting.

**Design:**

This cross-sectional survey was a one-stage cluster random sample of 80 hospitals in four South African provinces, selected with stratification from the public and private health sectors. On the survey day, all nurses working in critical care, theatre, emergency, maternity, and general medical and surgical wards completed a self-administered questionnaire after giving informed consent. In addition to demographic information, the questionnaire elicited information on the frequency of agency nursing, moonlighting, and overtime, and the nurses’ reasons for doing moonlighting. Survey data were weighted and analysed using STATA version 12.

**Results:**

The majority of survey participants (*n*=3,784) were South African (98.0%), female (92.7%), and employed in government (52.8%). Their mean age was 41.5 years (SD 10.4). The occurrence of moonlighting among nurses in the 12 months preceding the survey was 28.0% [95% CI: 24.2–32.1], the frequency of agency nursing was 37.8% [95% CI: 32.4–43.6], while 56.0% of nurses did overtime [95% CI: 51.4–60.4]. In the multiple logistic regression analysis, predictors of moonlighting were province, sector of primary employment, unit of work, category of nurse, and having children. The odds of moonlighting was 1.51 [95% CI: 1.03–2.21] times higher for private sector nurses than for public nurses, while the odds ratio for auxiliary nurses was 0.61 [95% CI: 0.47–0.79] compared to professional nurses. The odds of moonlighting was 1.49 [95% CI: 1.18–1.89] for nurses with children, compared to those without.

**Conclusions:**

Agency nursing, moonlighting, and overtime are common among South African nurses, but have received insufficient policy attention. These issues need to be addressed as part of the implementation of comprehensive health workforce strategies.

Human Resources for Health (HRH) are critical to good health outcomes and improved health systems functioning (1). In South Africa, the public sector faces a major challenge to produce, recruit, and retain scarce categories of health professionals (2, 3). Nurses make up the largest group of health care providers in the country. The nurse workforce consists of professional nurses (4 years of training), enrolled nurses (2 years of training), and nursing assistants or auxiliaries (1 year of training). In 2010, there were an estimated 81,925 public sector vacancies for all categories of nurses (2). Hence, the HRH crisis in the country is synonymous with the nursing crisis (4). The optimal performance of the health system is dependent on addressing nursing challenges (5).

The key dimensions of the nursing crisis in South Africa are well-recognised and include the following: inadequate production; high vacancy rates, coupled with an increased use of temporary agency nurses; international migration; insufficient staff in rural areas; an ageing workforce, low staff morale, and sub-optimal performance, exacerbated by widespread reports of moonlighting (3, 4). Although the recognition of the nursing crisis represents an important advance, information gaps remain. Anecdotal evidence suggests an increase in moonlighting among nurses and in the use of temporary agency nurses in the health sector, with many reported negative consequences for health care delivery. Knowledge on moonlighting and agency nursing is important for several reasons: it provides an indication of the time and financial pressures faced by individual health workers; it enhances understanding of the health system consequences of these phenomena; and it can assist with shaping appropriate health sector policies to deal with the challenges of equity and health care provision (1, 6–8).

*Moonlighting*, also known as multiple job-holding, is commonly understood as having at least one additional job, in addition to a primary full-time job (7). Much of the research on moonlighting has been done in high-income countries (8–11), and by labour economists, drawing on the economic theory of labour supply that views moonlighting activity primarily as a source of income (7, 9, 12, 13). A few studies that have examined moonlighting among medical doctors (14–16) suggest that doctors also do moonlighting in order to interact with fellow professionals in a health practice site, get approval from peers, or to enhance their knowledge and skills, thus supporting the sociological theories related to professions (7). *Organisational theories* of moonlighting suggest that although moonlighting provides workers with additional income, training, and other benefits, it could also change their perceptions, decisions, and behaviours at their primary jobs (9, 17). The latter could impact on their performance at their primary jobs and influence both absenteeism and turnover (9, 17). A variation of this theory is one that is related to the role of a hierarchical organisation or bureaucracy on individuals’ motivations, tasks and performance (7). Moonlighting, if done as part of a public service job, is seen as the breakdown of the bureaucratic work model (7, 18).

In *governance theory*, moonlighting is seen as an indicator of poor governance in health service delivery, and as a form of corruption that affects provider–patient interactions and health system performance (19–21).

Notwithstanding the reported problems of moonlighting in the health sector, there are few studies from low- and middle-income countries (LMICs) (7, 8, 22–24). Although these studies concluded that moonlighting is widespread, there is little quantitative evidence at country level or in the health system on the extent or characteristics of moonlighting, the reasons for doing so and the consequences for the use of scarce public health resources (7, 8). In South Africa, evidence on moonlighting among nurses is scant, except for a superficial investigation by the Public Service Commission (25) and a small qualitative study to describe critical care nurses’ reasons for and experiences of doing moonlighting (26).

Similarly in the case of agency nursing, much of the existing literature is concerned with high-income countries (27–30). Agency nursing is a form of casual employment (31), typically done through a temporary employment service provider (the nursing agency) who employs the nurse and then contracts the nurse to a public or private health facility that needs the nursing service. It involves a triangular form of employment that includes a third party (the nursing agency) who is an intermediary between the employee (the nurse) and the employer (public or private health authority). In the triangular employment, there is no formal relationship between the employee (the nurse) and employer (the health authority). The 1978 South African Nursing Act (section 1) defines a nursing agency as *a business which supplies registered nurses or midwives or enrolled nurses or nursing auxiliaries to any person, organisation or institution, whether for gain or not and whether in conjunction with any other service rendered by such business or not* (32: 4). A key focus of the limited literature on agency nursing has been on the management of the agency nurses. The few empirical studies tend to be descriptive, often lacking the methodological rigour needed to make generalisable conclusions (27–30). There is a similar dearth of literature on agency nursing in South Africa, and in sub-Saharan Africa.

In the majority of countries, including South Africa, the maximum working hours and minimum rest and break periods for formal sector employees are regulated (33). Knowledge on overtime among nurses is important as it affects their performance, an aspect identified as a gap in the global discourse on HRH (1). Furthermore, the occurrence of overtime is important in the context of agency nursing or moonlighting, as it also provides an additional or alternative source of income.

In light of major, impending health care reforms in South Africa towards universal coverage, an increasing emphasis on the role and performance of nurses, and limited empirical evidence, this paper examines the occurrence of agency nursing, moonlighting and overtime and the factors influencing moonlighting among nurses in South Africa.

## Methods

A cross-sectional nurses’ survey was conducted in the four South African provinces of the Eastern Cape (predominantly rural), Free State (mixed urban and rural), Gauteng (urban), and the Western Cape (predominantly urban) between November 2009 and April 2010. The selection of provinces was influenced by the location of commercial nursing agencies, the number of practising nurses, and financial and logistical considerations. The study was approved by the Human Research Ethics Committee of the University of the Witwatersrand, Johannesburg, and the relevant public and private health care authorities. All participants provided written, informed consent.

In each of the four provinces, the sampling frame consisted of all public and private hospitals. In the case of the public sector, the hospitals were stratified by type of hospital: central, regional, district, and specialised (e.g. tuberculosis). A stratified random sample of public sector hospitals was then selected from each stratum. In the case of the private sector, the hospitals were divided by ownership category into those with less than 100 beds, and those with 100 or more beds. A stratified random sample of private sector hospitals was then selected in each ownership category. The number of public or private hospitals selected from each stratum was therefore proportional to the total number of hospitals in that stratum.

This study was therefore a weighted one-stage cluster randomised sample stratified by province and sector. Hospitals were considered as clusters. Eighty hospitals were selected in the four provinces, half in the public sector, and half in the private sector. Within each sampled hospital, all nurses working in critical care, emergency, operating theatre, maternity and general medical or surgical wards were approached on the 24-hour survey day for voluntary participation. All respondents were assured of confidentiality.

Once a nurse had agreed to participate and after providing informed consent, he/she completed a self-administered questionnaire. In addition to demographic information, the questionnaire focused on the occurrence of agency nursing, moonlighting and overtime in the preceding 12-months, and reasons for moonlighting or agency nursing, using a seven-point Likert scale (strongly disagree to strongly agree). These reasons were identified in the international literature and in the formative research conducted prior to the survey.

In the study, *agency nursing* was defined as any accredited nurse providing temporary cover in a hospital and paid for by a commercial nursing agency. *Moonlighting* was defined as *additional paid work*—whether of a nursing or non-nursing nature—done concurrently by nurses in a private health facility, another government health facility, an insurance company, private health laboratory or in the same health care facility while holding a primary, paid nursing job, but excluding overtime. *Overtime* was defined as additional paid work performed at the primary employer for extra hours over and above the normal working day or week and paid for by the primary employer. The questionnaire took between 10 and 15 min to complete. Each nurse received a non-monetary gift to the value of R30 (around US$4) to thank them for their participation in the survey.

Survey data were analysed using STATA^®^ 12. All analyses were weighted to reflect the population distribution of different categories of nurses between, the public and private health sectors, and the four study provinces. We also adjusted for the clustering and stratification introduced by the cluster sampling design. Univariate logistic regression models were fitted to find factors which were associated independently with moonlighting. Only factors associated with moonlighting that were found to be statistically significant were considered in the model building exercise using a multiple logistic regression model. All statistical tests were carried out at 5% significance level.

## Results

The study recruited 3,803 participants and after excluding ineligible groups (student nurses and hospital care workers), this resulted in a sample of 3,784 nurses. An overall response rate of 90% was obtained in the survey.

### Participant characteristics

The age of participants ranged from 19 to 71 years, with a mean age of 41.5 years (standard deviation 10.4), with little provincial or sectoral variation. The majority of participants were South African (98.0%), female (92.7%), black African (54.2%), and 52.8% were employed in provincial government (Table 1).

**Table 1:** Demographic and employment characteristics of survey participants

Characteristic	Gauteng	Eastern Cape	Western Cape	Free State	Total
Mean age (Standard Deviation)	39.4 (10.5)	43.4 (10.4)	42.2 (10.4)	42.1 (9.8)	41.5 (10.5)
Age group					
< 25 years (%)	62 (5.5)	25 (3.3)	44 (4.6)	19 (2.3)	150 (4.1)
25–34 years (%)	355 (31.2)	157 (20.6)	196 (20.6)	180 (21.8)	888 (24.1)
35–44 years (%)	354 (31.1)	201 (26.3)	271 (28.5)	286 (34.6)	1,112 (30.2)
45–54 years (%)	265 (23.3)	267 (35.0)	322 (33.9)	261 (31.6)	1,115 (30.3)
55+ years (%)	101 (8.9)	114 (14.9)	118 (12.4)	80 (9.7)	414 (11.2)
Sex					
Female (%)	1,099 (91.4)	736 (95.0)	921 (96.1)	733 (88.3)	3,489 (92.7)
Male (%)	103 (8.6)	39 (5.0)	37 (3.9)	97 (11.7)	276 (7.3)
Race					
Black African (%)	955 (80.5)	408 (52.8)	197 (20.6)	466 (56.3)	2,026 (54.2)
Coloured (%)	59 (5.0)	199 (25.8)	522 (54.7)	70 (8.5)	850 (22.7)
Indian (%)	12 (1.0)	12 (1.6)	7 (0.7)	1 (0.1)	32 (0.9)
White (%)	160 (13.5)	153 (19.8)	229 (24.0)	291 (35.1)	833 (22.3)
Marital status					
Married (%)	476 (39.8)	370 (47.7)	445 (46.4)	401 (48.4)	1,693 (45.0)
Living together (%)	58 (4.9)	10 (1.3)	34 (3.6)	28 (3.4)	130 (3.5)
Single (%)	512 (42.7)	279 (36.0)	308 (32.1)	229 (27.6)	1,328 (35.3)
Divorced/Separated (%)	94 (7.9)	67 (8.6)	128 (13.4)	121 (14.6)	410 (10.9)
Widowed (%)	57 (4.8)	50 (6.4)	44 (4.6)	50 (6.0)	201 (5.3)
Children					
Median number of children (range)	2 (1–7)	2 (1–14)	2 (1–6)	2 (1–6)	2 (1–14)
Median age of youngest child	10	14	14	12	12
Nursing category					
Professional nurse (%)	632 (53.4)	413 (54.2)	424 (45.1)	441 (53.5)	1,910 (51.5)
Enrolled nurse (%)	294 (24.8)	157 (20.6)	217 (23.1)	150 (18.2)	818 (22.1)
Auxiliary nurse (%)	258 (21.8)	192 (25.2)	299 (31.8)	233 (28.3)	982 (26.5)
Median years qualified as a nurse (mean)	10 (12.7)	20 (17.4)	20 (17.9)	16 (16.6)	15 (15.9)
Primary job					
Provincial government (%)	728 (62.0)	399 (52.3)	487 (51.4)	341 (41.8)	1,955 (52.8)
Private sector (%)	323 (27.5)	314 (41.2)	367 (38.8)	396 (48.5)	1,400 (37.8)
Nursing agency (%)	124 (10.6)	50 (6.6)	93 (9.8)	79 (9.7)	346 (9.4)
Median years at primary job (mean; range)	6 (8.9; 1–45)	7 (11.5; 1–44)	8 (11.2; 1–47)	8 (10.2; 1–44)	7 (10.3; 1–47)
Unit of work					
Paediatric critical care (%)	81 (6.9)	17 (2.3)	81 (8.8)	4 (0.5)	183 (5.1)
Adult critical care (%)	162 (13.9)	87 (12.0)	78 (8.5)	94 (12.6)	421 (11.8)
High care (%)	8 (0.7)	15 (2.1)	5 (0.5)	5 (0.7)	33 (0.9)
Theatre (%)	194 (16.6)	139 (19.2)	125 (13.6)	210 (28.2)	668 (18.8)
Emergency (%)	115 (9.9)	91 (12.5)	124 (13.5)	62 (8.3)	392 (11.0)
Maternity (%)	204 (17.5)	70 (9.6)	193 (21.0)	107 (14.4)	574 (16.1)
General wards (%)	346 (29.5)	262 (36.1)	312 (33.9)	221 (29.7)	1,140 (32.0)
Psychiatry (%)	54 (4.6)	24 (3.3)	1 (0.1)	38 (5.1)	117 (3.3)
Outpatient department (%)	5 (0.4)	21 (2.9)	1 (0.1)	3 (0.4)	30 (0.8)

### Moonlighting and agency nursing among nurses

**Fig. 1:**
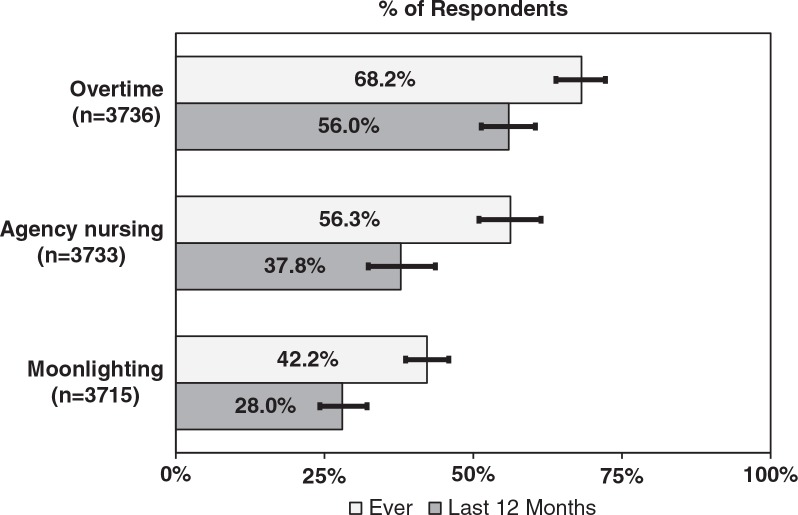
Prevalence of overtime, agency nursing or moonlighting.

Figure 1 shows the occurrence of agency nursing, moonlighting and overtime. The occurrence of moonlighting among nurses in the 12 months preceding the survey was 28.0% [95% CI: 24.2–32.1], the frequency of agency nursing was 37.8% [95% CI: 32.4–43.6], while 56.0% of nurses did overtime [95% CI: 51.4–60.4]. A significant majority (69.2% [95% CI: 64.1–73.8]) of respondents had done overtime, moonlighting or agency nursing in the year preceding the survey.

**Fig. 2:**
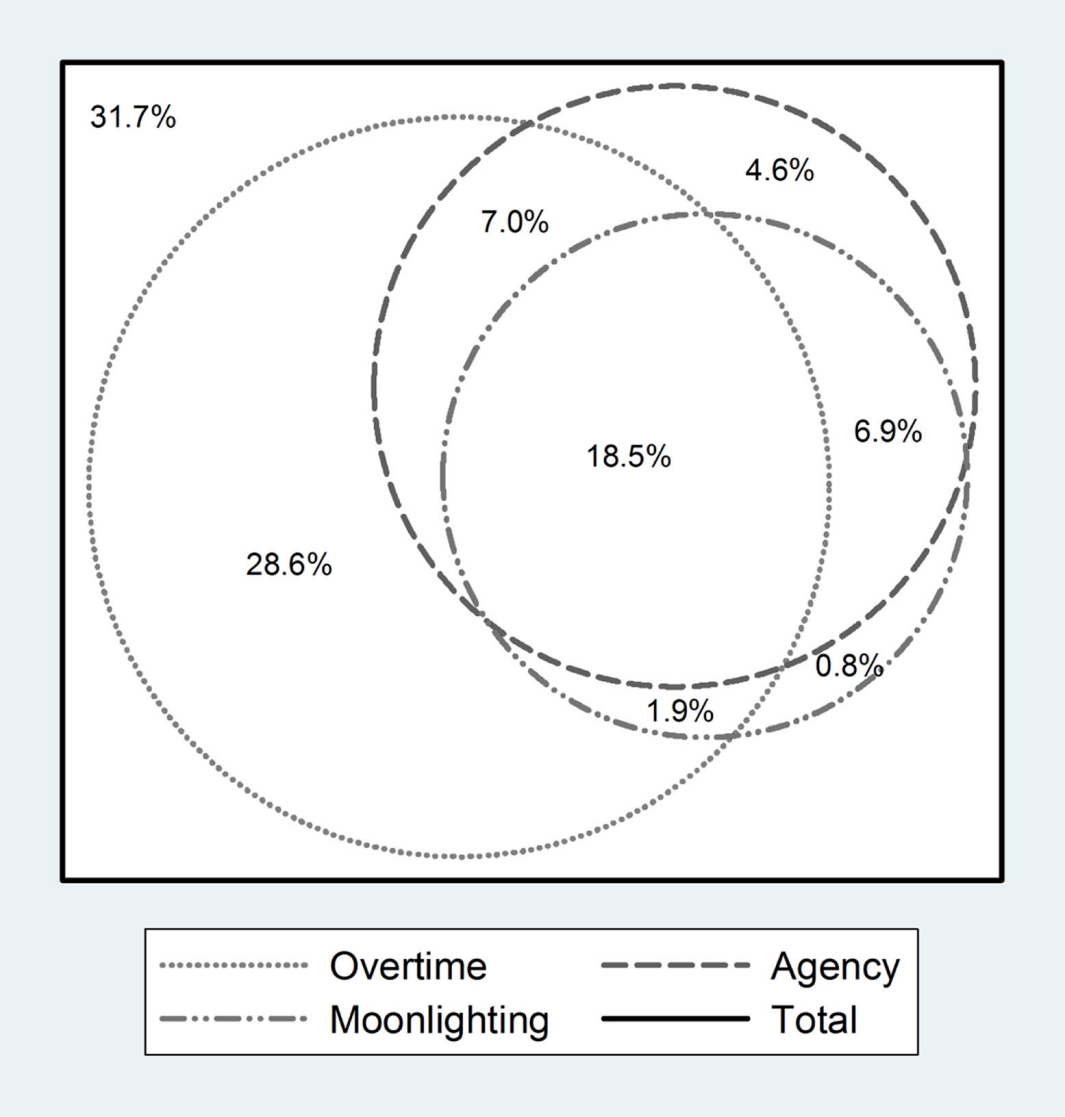
Relationship among agency nursing, moonlighting and overtime.

Figure 2 shows the relationship between reported agency nursing, moonlighting and overtime in the year preceding the survey, and the different permutations of agency nursing, moonlighting or overtime that participants could engage in. As can be seen from Fig. 2, 31.7% of participants had not done overtime, moonlighting or agency nursing. A significant proportion (18.5%) of participants reported all three activities in the year preceding the survey—28.6% of participating nurses had done only overtime, 0.8% did only moonlighting and 4.6% had only done agency nursing.

Table 2 shows the prevalence of agency nursing, moonlighting and overtime in the year preceding the survey for the four surveyed provinces, by primary employer (provincial government, private health sector, or nursing agency) and by nursing category (professional nurse, enrolled nurse or nursing auxiliary/assistant). Statistically significant differences between groups using the Chi-square test are shown in the table.

**Table 2:** Reported overtime, agency nursing or moonlighting in the preceding year

Variable	%	Overtime	%	Agency nursing	%	Moonlighting
Overall	56.0		37.8		28.0	
Sex						
Male	53.9	NS	36.0	NS	31.1	NS
Female	56.1		37.9		27.7	
Age group						
< 25 years	34.0		34.8		23.5	
25–34 years	52.3		41.4		30.1	
35–44 years	60.9	***	43.4	***	33.1	***
45–54 years	57.5		33.6		25.8	
55+ years	55.4		24.6		15.8	
Province						
Gauteng Province	58.1		53.6		37.1	
Eastern Cape	44.2	**	11.9	***	11.1	***
Western Cape	60.2		37.9		28.7	
Free State	69.2		33.1		30.3	
Primary employer						
Provincial government	54.7		28.4		24.2	
Private health sector	68.7	***	58.4	***	40.6	***
Nursing agency	35.6		79.4		34.1	
Nursing category						
Professional nurse	57.9		39.6		32.7	
Enrolled nurse	57.1	NS	40.9	*	28.9	***
Auxiliary nurse/ENA	52.5		33.4		20.5	
Work unit						
Paediatric critical care	61.7		64.4		38.2	
Adult critical care	63.0		72.4		59.0	
Theatre	66.1	**	32.6	***	24.9	***
Emergency	60.2		33.6		23.9	
Maternity	56.6		42.6		31.9	
General wards	49.2		31.7		23.3	

NS: Non-significant

* *p*<0.05

** *p*<0.01

*** *p*<0.001 (Chi-squared test).

As can be seen from Table 2, 56.0% of nurses reported overtime in the year preceding the survey. Overtime rates were highest in the Free State Province, the private sector, among professional nurses, and for nurses working in theatre. For agency nursing, Gauteng Province topped the list with 53.6% of nurses reporting agency nursing. Surprisingly, the agency nursing rate was higher among private sector nurses (58.4%) compared to provincial government nurses (28.4%), and for enrolled nurses (40.9%) compared to professional (39.6%) or auxiliary nurses (33.4%). The prevalence of moonlighting among nurses working for the private health sector (40.6%) was also higher in comparison to provincial government nurses (24.2%). Critical care nurses reported higher moonlighting rates (38.2% paediatric critical care; 59.0% adult critical care) reflecting high demand for skilled nursing care in both the public and private health sectors. High rates of moonlighting were also found in maternity units (31.9%), but relatively low rates in theatre (24.9%) and emergency units (23.9%).

### Reported reasons for moonlighting

Table 3 shows the participants reported reasons for moonlighting ranked in order of importance by mean score (out of 7), and also indicates the percentage of moonlighting respondents who agreed with each statement.

**Table 3:** Reasons for moonlighting in 12 months preceding survey

Reason	Mean score	% Agreeing
Taking care of patients	6.3	92.7
Opportunity to learn new nursing skills	6.0	87.8
Relationship with co-workers	5.8	84.4
Agency's weekly pay	5.7	81.8
Choice of unit/ward	5.3	75.4
Do it for the money	5.2	72.5
Job variety	5.2	74.9
Stimulating work	5.1	71.2
Quality of supervision	5.0	68.5
Feedback on quality of work	5.0	68.1
Modern equipment/infrastructure	5.0	67.7
Selection of working hours	4.8	65.1
Money owed to revenue service	3.1	29.2

As can be seen from Table 3, both financial reasons (more money and weekly agency pay); as well as non-financial reasons (choice of unit/ward; job variety; opportunity to learn new nursing skills; taking care of patients; and relationship with co-workers) featured strongly as reasons for moonlighting among nurses.

### Predictors of moonlighting

In the multiple logistic regression analysis, predictors of moonlighting were province, sector of primary employment, unit of work category of nurse, sex and having children (Table 4). In the logistic regression, the odds of moonlighting were 0.29 [95% CI: 0.15–0.55] for nurses working in the Eastern Cape Province, compared to nurses in Gauteng Province, and this was significant (*p*<0.001). The odds of moonlighting were 0.85 [95% CI: 0.52–1.38] for nurses working in the Free State Province, compared to nurses in Gauteng Province.

**Table 4:** Predictors of moonlighting in the previous year

	Variable	Odds Ratio	[95% CI]	*p*	Sig
Sex	Male	–	–	–	
	Female	0.74	[0.51; 1.08]	0.117	
Age group	<25 years	–	–	–	
	25–34 years	1.33	[0.69; 2.54]	0.389	
	35–44 years	1.29	[0.64; 2.58]	0.467	
	45–54 years	1.13	[0.58; 2.21]	0.713	
	55+ years	0.61	[0.30; 1.22]	0.158	
Marital status	Married/living together	–	–	–	
	Single	1.13	[0.95; 1.34]	0.175	
	Divorced/separated	1.03	[0.80; 1.31]	0.836	
Any children	No	–	–	–	
	Yes	1.49	[1.18; 1.89]	0.001	***
Province	Gauteng	–	–	–	
	Eastern Cape	0.29	[0.15; 0.55]	<0.001	***
	Western Cape	0.77	[0.50; 1.20]	0.240	
	Free State	0.85	[0.52; 1.38]	0.498	
Sector	Provincial government	–	–	–	
	Private sector	1.51	[1.03; 2.21]	0.037	*
	Nursing agency	1.11	[0.71; 1.72]	0.655	
Nursing category	Professional nurse	–	–	–	
	Enrolled nurse	0.83	[0.59; 1.16]	0.262	
	Nursing assistant/auxiliary nurse	0.61	[0.47; 0.79]	<0.001	***
Unit of work	Paediatric critical care unit	–	–	–	
	Adult critical care unit	2.13	[1.24; 3.68]	0.007	**
	Theatre	0.67	[0.42; 1.06]	0.088	
	Emergency	0.64	[0.39; 1.07]	0.091	
	Maternity	0.89	[0.58; 1.38]	0.606	
	General wards	0.62	[0.40; 0.97]	0.035	*
	Other	0.30	[0.12; 0.78]	0.013	*
Constant		0.59	[0.33; 1.08]	0.085	

* Significance: *p*<0.05

** *p*<0.01

*** *p*<0.001.

The odds of moonlighting was 1.51 [95% CI: 1.03–2.21] times higher for private sector nurses than for public nurses, while the odds ratio for auxiliary nurses was 0.61 [95% CI: 0.47–0.79] compared to professional nurses. Females appeared less likely than males to do moonlighting, with the odds being 0.74 [95% CI: 0.51–1.08] for female nurses compared to male nurses, although this difference was not statistically significant. The odds of moonlighting was 1.49 [95% CI: 1.18–1.89] for nurses with children, compared to those without children.

## Discussion

This is the first, large cross-sectional survey on the prevalence of agency nursing, moonlighting and overtime among nurses in South Africa, and indeed in sub-Saharan Africa. We found that 37.8% of respondents had done agency nursing. Our study findings are similar to a 2008 Australian workforce review that found that 49.8% of the nursing workforce consisted of part-time and casual nurses (34). In 2013, a newspaper survey of 100 hospital trusts in the UK found that temporary nursing costs have increased by around 20%, a reflection of the increase in the utilisation of agency nurses (35).

In our survey, 28.0% of nurses had done moonlighting in the year preceding the survey. We could not find comparable, large-scale, studies on agency nursing or moonlighting in other LMICs, or even in high-income countries. In the USA, the official average moonlighting rate among the labour force was 5.2% in 2004, while in the UK, it is estimated that between 8 and 10% of the UK labour market engage in moonlighting (17).

Nonetheless, empirical studies have found higher moonlighting rates compared to government statistics, which are collected in high-income countries. A 2001 postal survey among teachers in one state in the USA found a moonlighting frequency of 34% (17). Other studies have found that the frequency of moonlighting among medical specialists in training varies from 31 to 50%, with considerable variation by medical speciality (11, 14, 36). Notwithstanding the differences in methodology and the nature of the occupations studied, these moonlighting prevalence figures are comparable to our study findings. Of concern is that 69.2% of nurses had done overtime, moonlighting or agency nursing in the previous year. This implies that these nurses work excessive hours, and this has been shown to impact negatively on patient care (37–39).

Our study found statistically significant differences in the prevalence of nurses’ overtime, moonlighting or agency nursing by geographical area (province), primary employer, category of nurse and work unit (Table 2). The differences were confirmed by multiple regression analysis (Table 4). Critical care nurses reported higher moonlighting rates reflecting high demand for skilled nursing care in both the public and private health sectors. In contrast, our study found relatively low frequency of moonlighting among theatre and emergency unit nurses, which may reflect the difficulty of bringing in casual nurses into a setting which requires familiarity and in-depth knowledge of surgeons’ preferences (theatre) or dealing with complex emergencies. Studies among medical specialists in training have also found that the prevalence of moonlighting varies by area of specialty (11, 14, 36, 40).

In contrast to other studies, our study found no differences in agency nursing, moonlighting or overtime between male and female nurses. A 2001 study among teachers in the USA found that male teachers had a higher moonlighting rate (46%) compared to female teachers (30%) (17). The US study found that the spouses of male teachers earned lower wages compared to the spouses of female teachers, indicating lower total household income and possibly greater financial need (17). Another study also found gender differences in moonlighting (6).

Our survey found that the non-pecuniary reasons such as: taking care of patients (92.7%); the opportunity to learn new nursing skills (87.8%); and relationships with co-workers (84.4%) also played a role in the moonlighting decisions of nurses. These findings on the multiple and varied reasons for moonlighting are supported by the findings of other studies (7, 8, 22, 27). Studies in the United States on the moonlighting determinants among physicians during their residency training periods found that economic and professional factors influenced decisions about a second job (10, 11).

In this survey, 72.5% of nurses agreed that more money was a factor and 81.8% agreed that weekly agency pay was a reason, thus supporting the general findings of labour economists and among medical residents that one of the main reasons for moonlighting is economic or financial (6, 12, 14, 16, 36).

This article provides empirical evidence on the reasons for and predictors of moonlighting at the national level in South Africa. The study relied on nurses’ self-reported information which is subject to socially desirability bias. This could mean that participants under-reported moonlighting or downplayed the financial reasons for moonlighting. However, the self-administered questionnaire allowed for greater privacy and is likely to have led to more accurate reporting of moonlighting, overtime and temporary agency nursing. The high response rate obtained in the survey is a major strength, thus eliminating the problem of non-response bias typical of surveys of this nature.

Hitherto, the concepts of agency nursing and moonlighting have been poorly understood and under-researched in South Africa. The main rationale for the survey was to begin to address the knowledge gaps and provide evidence that will help shape the policy on broader processes of nursing casualisation, but specifically on nursing agencies and moonlighting in South Africa. This study makes an important contribution to improved knowledge and understanding of agency nursing, moonlighting and overtime among nurses in South Africa. As is the case with many health workforce studies, the information in this paper is based on cross-sectional rather than longitudinal data. Hence, the survey only presents the prevalence of overtime, agency nursing or moonlighting at a point in time. We could not verify the reported reasons for temporary agency nursing or moonlighting and did not determine the association between agency nursing and staff shortages, or between moonlighting and objective measures of financial need. We also did not determine issues around nurses’ productivity and the relationship to overtime or agency nursing.

There are important policy implications of our study. Our study indicates that moonlighting and agency nursing are widespread in the South African health system, with many nurses working excessively long hours. As was pointed out by the 2013 report of the Global Health Workforce Alliance, moonlighting possibilities are driven by the nature of the health worker labour market (1). Because rural or poor areas (such as the Eastern Cape Province) offer little prospect of moonlighting compared with more affluent urban areas, moonlighting is an important contributor to geographical mal-distribution. There appears to be a crisis in critical care units with high reliance on agency nursing, while operating theatres rely on excessive staff overtime. The latter leads to exhaustion and possible burnout, both impacting on the quality of patient care and the performance of health care providers (37–39). The reported reasons for moonlighting are complex, and included both financial and non-financial factors. In the short-term, interventions must include improved management of nursing agencies; an open dialogue and debate on moonlighting in the South African health system, and its implications for ethical and accountable nursing practice; and improved staff scheduling. The findings also point to the need for improved performance management of all staff, including nurses. The non-financial reasons for moonlighting such as recognition and appreciation of nurses, equitable training opportunities, and supportive management and supervision could be addressed without additional money or resources in the health system. In the long-term, these issues need to be addressed as part of the implementation of comprehensive health workforce strategies.

## Conclusion

Agency nursing and moonlighting among nurses are common but these issues have received insufficient policy and scholarly attention in South Africa (and internationally). As a result of the study, the South African government has identified the management of moonlighting and the regulation of commercial nursing agencies as important policy priorities, and this is reflected in the 5-year HRH strategy for the health sector (2). In the long-term, improving human resource planning, the development of national nursing norms and standards and the revision of nurses’ salaries and working conditions and on-going monitoring of agency nursing, moonlighting and overtime will give effect to the goals of the HRH strategic plan. Action on the findings of the study is of particular importance in light of the global emphasis on universal health coverage, and the importance of the health workforce in reaching this ambitious goal.
